# Preoperative imaging for endometriosis awareness: an online survey study

**DOI:** 10.3389/frph.2026.1917458

**Published:** 2026-09-03

**Authors:** Bassem Gerges, Allie Eathorne, George Condous

**Affiliations:** 1 Acute Gynaecology, Early Pregnancy and Advanced Endosurgery Unit, Sydney Medical School Nepean, Nepean Hospital, University of Sydney, Kingswood, NSW, Australia; 2 Sydney West Advanced Pelvic Surgery (SWAPS), Blacktown Hospital, Blacktown, NSW, Australia; 3 Medical Research Institute of New Zealand, Wellington, New Zealand

**Keywords:** awareness, computed tomography, endometriosis, preoperative imaging, magnetic resonance imaging, preoperative diagnosis, ultrasound

## Abstract

**Introduction:**

In 2024, an international consensus statement on non-invasive imaging techniques for the diagnosis of pelvic deep endometriosis was published, recommending transvaginal sonography as the first-line imaging modality because of cost, availability, and low impact on the environment. Despite the increasing body of evidence supporting the use of imaging in the preoperative diagnosis of endometriosis, there is a paucity of data to confirm patient awareness. The aim of this study was to use an online survey or questionnaire from patients to collect quantitative data regarding their awareness, exposure, and experience with preoperative imaging for the diagnosis of endometriosis.

**Methods:**

This was a cross-sectional study, based on an online, international survey completed by patients with self-reported endometriosis from multiple online endometriosis groups. The survey went live from November 2025 to March 2026 and consisted of women and gender-diverse individuals with self-reported endometriosis, with a minimum required number of 100 participants.

**Results:**

A total of 180 participants started the survey, of which 142 (78.9%) completed the exercise. The mean age of the participants was 31.6 [standard deviation (SD) 8.7] years, with a median time of endometriosis diagnosis of 2 years (interquartile range 1–5), of whom 99 (69.7%) had at least one surgery, with a mean number of surgeries of 1.8 (SD 1.2). The majority of participants were from Australia (66.2%), followed by Canada (16.9%), New Zealand (11.3%), and the United Kingdom (2.1%), and an equal number were from Brazil, India, the Philippines, and Spain (0.7% each). Overall, 53.5% of participants agreed that endometriosis can be diagnosed without surgery, while only 32.4% agreed that bowel endometriosis can be diagnosed before surgery. Of these, the percentages of participants who agreed that bowel endometriosis can be preoperatively diagnosed with ultrasound, magnetic resonance imaging, computed tomography, and colonoscopy were 32%, 35%, 15%, and 15%, respectively. The participants obtained knowledge about endometriosis investigations and treatments from various sources, including their gynecologists (71.8%), general practitioners (57%), fertility specialists (11.3%), family members (12%), friends (21.8%), social media (68.3%), and other sources (29.6%).

**Conclusion:**

There is a disparity between participant awareness in our study and the evidence in the literature regarding the availability and accuracy of preoperative imaging for the diagnosis of deep endometriosis, possibly due to the misinformation disseminated via social media**.**

## Introduction

Endometriosis is typically characterized by the deposition of endometrial-like cells outside the uterine cavity, resulting in lesions ranging from peritoneal implants to deep endometriosis (DE). DE is defined as endometrial implants or nodules of ≥5 mm subperitoneal infiltration, which can involve multiple organs in the pelvis, including the bladder, vagina, uterosacral ligaments, bowel, and peritoneum (1,2). A resultant distortion in anatomy can occur, including obliteration of the pouch of Douglas or *cul-de-sac*. Endometriosis can cause symptoms of chronic pelvic pain, dyspareunia, urinary and/or bowel symptoms, and infertility (3–5). This group of women requires both gynecological and colorectal input at surgery. Preoperative imaging is being increasingly used to predict the level of complexity of laparoscopic surgery for endometriosis. It has the potential to facilitate triaging women with suspected bowel DE to the most appropriate surgical expertise required for laparoscopic intervention for endometriosis.

In 2016, the International Deep Endometriosis Analysis (IDEA) Group (6) published a consensus statement to standardize the terms, definitions, and sonographic evaluation of the pelvis and identified five locations for examination, namely, the bladder, the rectovaginal septum, the posterior vaginal fornix, the uterosacral ligaments/torus uterinus, and the rectum/rectosigmoid/sigmoid. Since then, there have been multiple systematic reviews and meta-analyses that have confirmed the diagnostic accuracy of various imaging modalities, with transvaginal ultrasound and magnetic resonance imaging (MRI) being quite comparable (7–12). More recently, in 2024, an international consensus statement on non-invasive imaging techniques for the diagnosis of pelvic DE, involving seven international societies and simultaneously published in five journals, recommended transvaginal ultrasound as the first-line imaging modality because of cost, availability, and low impact on the environment (13–17).

Despite the increasing body of evidence supporting the use of imaging in the preoperative diagnosis of endometriosis, there is a paucity of data to confirm patient awareness. The aim of this study was to use an online survey or questionnaire from patients to collect quantitative data (18) regarding their awareness, exposure, and experience with preoperative imaging for the diagnosis of endometriosis. The advantages of an online survey include the ability to target the desired audience, while saving time and cost (19).

## Methods

This was a cross-sectional study, based on an online, international survey completed by members with self-reported endometriosis of various online endometriosis groups, including Endometriosis Australia, Endometriosis New Zealand, Endometriosis Canada, Asociación de Endometriosis España, GAPENDI, and Endometriosis UK. The administrators of these groups were formally approached via email and permission obtained for the survey to be disseminated through their individual channels. The survey was an electronic questionnaire (Supplementary Appendix 1) distributed via the REDCap platform, which went live from November 2025 to March 2026, from which the data were analyzed. Ethics approval was obtained from the local Human Research Ethics Committee of Nepean Blue Mountains Local Health District (HREC 2025/ETH01032). Participants were informed about the essential elements of the research, including the risks and benefits of participation, on the intro page of the survey. Their decision to complete and submit the survey constituted informed consent.

### Study population

The study population consisted of all women and gender-diverse people with self-reported endometriosis who received the questionnaire and volunteered in completing the questionnaire, with a minimum of 100 participants.

The inclusion criteria were women and gender-diverse people aged above 18 years old with a self-reported diagnosis of endometriosis, while the exclusion criteria were women and gender-diverse people without a self-reported diagnosis of endometriosis.

### Statistical analysis

Continuous variables were summarized using the mean and standard deviation (SD), the median and the interquartile range (IQR), and minimum (min.) and maximum (max.). Categorical variables were summarized as counts and proportions expressed as percentages. Data descriptions were performed using SAS software, version 9.4.

## Results

A total of 180 participants started the survey, of whom 142 (78.9%) completed the exercise. The mean age of the participants was 31.6 (SD 8.7), with a median time of endometriosis diagnosis of 2 years (IQR 1–5), of whom 99 (69.7%) had at least one surgery, with a mean number of surgeries of 1.8 (SD 1.2) (Table 1). The majority of participants were from Australia (66.2%), followed by Canada (16.9%), New Zealand (NZ) (11.3%), the United Kingdom (2.1%), and an equal number from Brazil, India, the Philippines, and Spain (0.7% each). The most common symptoms were pelvic pain (95.1%), period pain (90.8%), bloating (89.4%), bowel symptoms (85.2%), and dyspareunia (72.5%). The diagnosis of endometriosis was based on surgical findings (71.8%), ultrasound (38%), and symptoms (33.1%). A total of 99 (69.7%) participants underwent surgery for endometriosis, of whom 7 (4.9%) had Stage I, 21 (14.8%) had Stage II, 23 (16.2%) had Stage III, 37 (26.1%) had Stage IV endometriosis, and 54 (38%) were unsure or not informed. These and the previous treatments trialed by all participants are detailed in Table 2, while Table 3 outlines the specific awareness of the various imaging modalities of all participants. Supplementary Tables S1 and S2 outline the previous treatments trialed and the awareness of the imaging modalities from participants by country, respectively.

**Table 1:** General characteristics of the respondents.

Variable	*N*	Mean (SD)	Median (IQR)	Min. to max.
Age (years)	141	31.6 (8.7)	30 (25–38)	17–53
How long ago was endometriosis diagnosed? (years)	142	3.9 (5.5)	2 (1–5)	0–31
Number of procedures	99	1.8 (1.2)	1 (1–2)	1–7
Endometriosis affecting fertility	142	6.5 (3.3)	7 (3–9)	1–10

**Table 2:** Details of endometriosis history.

Variable	*N* = 142 (%)
Relationship status	
Single	47 (33.1)
Engaged	13 (9.2)
Married	41 (28.9)
*De facto* (Two people living together as a couple on a genuine domestic basis without a marriage certificate)	35 (24.6)
Separated	2 (1.4)
Divorced	1 (0.7)
Other	3 (2.1)
Country/continent of origin	
Australia	94 (66.2)
Brazil	1 (0.7)
Canada	24 (16.9)
Spain	1 (0.7)
UK	3 (2.1)
Europe	1 (0.7)
NZ	16 (11.3)
Philippines	1 (0.7)
India	1 (0.7)
Highest level of education	
High school	29 (20.4)
Graduate certificate or diploma	36 (25.4)
Bachelor’s degree	51 (35.9)
Master’s degree	22 (15.5)
PhD	1 (0.7)
Other	3 (2.1)
Previously diagnosed with endometriosis = yes	134 (94.4)
Symptoms (multiple allowed)	
Period pain	129 (90.8)
Pelvic pain	135 (95.1)
Pain with intercourse	103 (72.5)
Bowel symptoms	121 (85.2)
Bladder symptoms	87 (61.3)
Bloating	127 (89.4)
Infertility	31 (21.8)
Other	38 (26.8)
Concerned about or have had trouble getting pregnant = yes	73 (51.4)
Previous IVF = yes	10 (7)
Trying to get pregnant for longer than 6 months = yes	22 (15.5)
Diagnosis method (multiple allowed)	
Based on symptoms	47 (33.1)
Surgery	102 (71.8)
Ultrasound	54 (38)
MRI	16 (11.3)
Other	12 (8.5)
Treatments tried (multiple allowed)	
Herbal = natural therapies	70 (49.3)
Diet	99 (69.7)
NSAIDs	121 (85.2)
Oral contraceptive pills	92 (64.8)
Progesterone-only pills	65 (45.8)
Ryeqo	8 (5.6)
Zoladex	7 (4.9)
Implanon	20 (14.1)
Mirena intrauterine device	70 (49.3)
Surgery	96 (67.6)
Other	16 (11.3)
Have not tried anything	4 (2.8)
Surgery for endometriosis = yes	99 (69.7)
Diagnosed endometriosis stage	
Stage 1	7 (4.9)
Stage 2	21 (14.8)
Stage 3	23 (16.2)
Stage 4	37 (26.1)
Unsure = Was not told	54 (38)

Percentages are based on *N* = 142, unless otherwise stated. Participants could select more than one response where indicated. CT, computed tomography; MRI, magnetic resonance imaging; NSAIDs, non-steroidal anti-inflammatory drugs; NZ, New Zealand; UK, United Kingdom.

**Table 3:** Patient understanding of preoperative imaging for the diagnosis of deep endometriosis.

Variable	*N* = 142 (%)
Preoperative investigations (multiple allowed)	
Blood tests	56 (39.4)
Ultrasound	131 (92.3)
MRI	41 (28.9)
Deep endometriosis ultrasound	43 (30.3)
CT scan	27 (19)
Other	7 (4.9)
Can endometriosis be diagnosed without surgery = yes	76 (53.5)
Can bowel endometriosis be diagnosed before surgery = yes	46 (32.4)
Bowel endometriosis diagnosis method (multiple allowed) (*N* = 46)	
Ultrasound	32 (69.6)
MRI	35 (76.1)
CT scan	15 (32.6)
Colonoscopy	15 (32.6)
Other	1 (2.2)
Referred for a deep endometriosis ultrasound = yes	56 (39.4)
Underwent a deep endometriosis ultrasound = yes	53 (94.6) *N* = 56
Reasons for not undergoing a deep endometriosis ultrasound (multiple allowed) (*N* = 3)	
Not available near me	0 (0)
Too expensive	2 (66.7)
Not convinced it was necessary	0 (0)
Other	1 (33.3)
Obtained knowledge about deep endometriosis ultrasound via (multiple allowed)	
Gynecologist	50 (35.2)
Fertility specialist	5 (3.5)
Family	1 (0.7)
Friend	3 (2.1)
Social media	10 (7)
Other	14 (9.9)
Did not have any before surgery	73 (51.4)
Deep endometriosis ultrasound can diagnose (multiple allowed)	
Stage I	38 (26.8)
Stage II	40 (28.2)
Stage III	55 (38.7)
Stage IV	78 (54.9)
Bowel nodules	52 (36.6)
Endometriomas	90 (63.4)
Adenomyosis	56 (39.4)
Other	33 (23.2)
MRI can diagnose (multiple allowed)	
Stage I	29 (20.4)
Stage II	35 (24.6)
Stage III	49 (34.5)
Stage IV	65 (45.8)
Bowel nodules	58 (40.8)
Endometriomas	87 (61.3)
Adenomyosis	53 (37.3)
Other	35 (24.6)
CT can diagnose (multiple allowed)	
Stage I	18 (12.7)
Stage II	15 (10.6)
Stage III	17 (12)
Stage IV	33 (23.2)
Bowel nodules	33 (23.2)
Endometriomas	68 (47.9)
Adenomyosis	30 (21.1)
Other	53 (37.3)
Sources of knowledge about endometriosis investigations obtained (multiple allowed)	
GP	81 (57)
Gynecologist	102 (71.8)
Fertility specialist	16 (11.3)
Family	17 (12)
Friends	31 (21.8)
Social media	97 (68.3)
Other	42 (29.6)

Percentages are based on *N* = 142, unless otherwise stated. Participants could select more than one response where indicated. CT, computed tomography; GP, general practitioner; MRI, magnetic resonance imaging.

**Figure 1:**
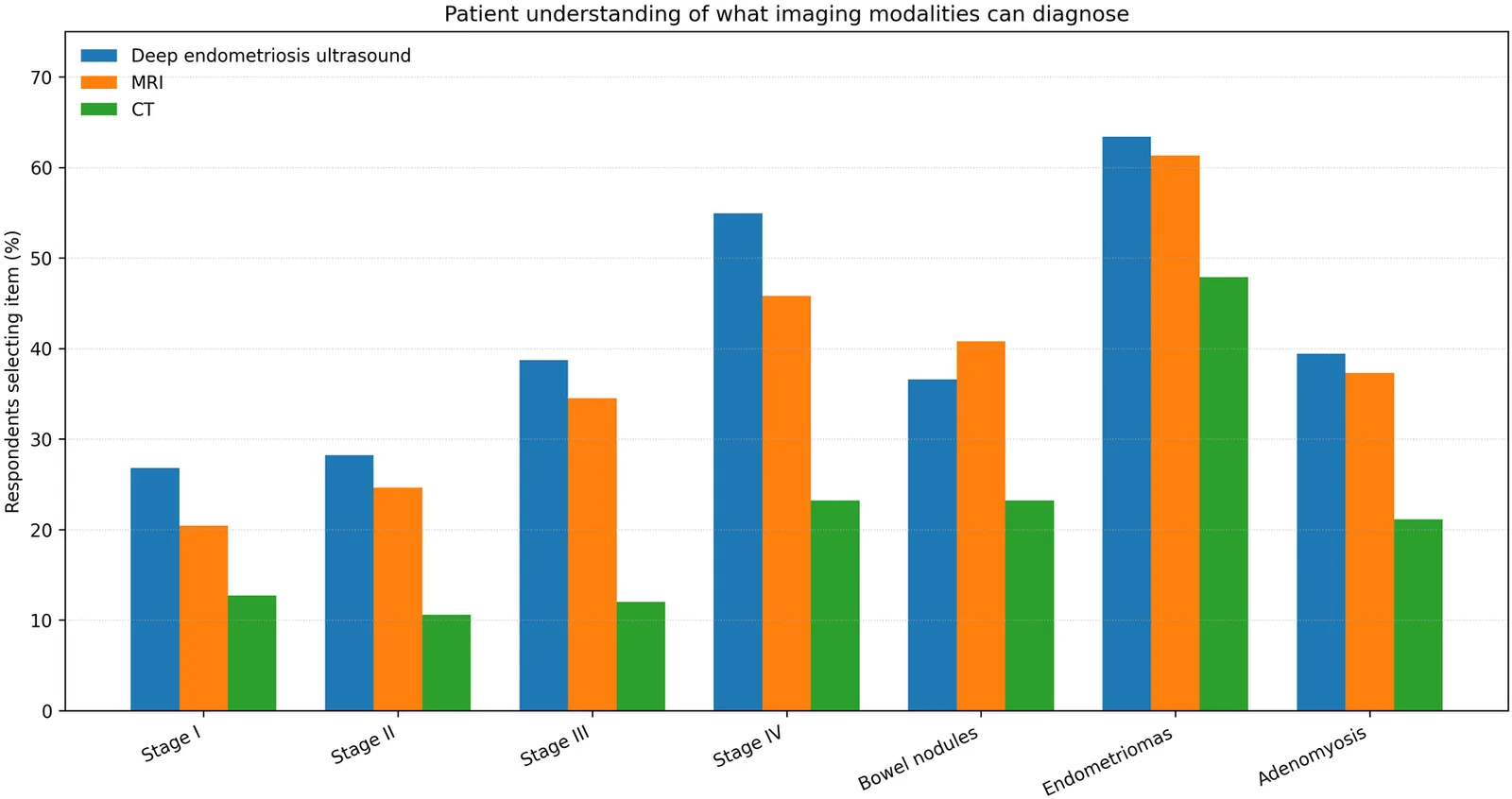
Patient understanding of imaging modalities. Respondent understanding of whether deep endometriosis ultrasound, MRI, and CT can diagnose endometriosis Stages I–IV, bowel nodules, endometriomas, and adenomyosis. CT, computed tomography; MRI, magnetic resonance imaging.

Overall, 53.5% of participants agreed that endometriosis can be diagnosed without surgery, while 32.4% agreed that bowel endometriosis can be diagnosed before surgery. Of the latter, the percentages of participants who agreed bowel endometriosis can be preoperatively diagnosed with ultrasound, MRI, computed tomography (CT), and colonoscopy were 32%, 35%, 15%, and 15%, respectively (Figure 1). Only 39.4% were referred for a DE ultrasound, of whom the majority (94.6%) had this performed. Over half of the participants (51.4%) did not have a preoperative DE, while the percentages of participants who obtained knowledge about DE ultrasounds from gynecologists, fertility specialists, family, friends, social media, and others were 50%, 5%, 1%, 3%, 10%, and 14%, respectively, of which “other” included Google and other specialists such as general practitioners, colorectal surgeons, and gastroenterologists. The participants obtained knowledge on endometriosis investigations and treatment from various sources, including their gynecologists (71.8%), general practitioners (57%), fertility specialists (11.3%), family (12%), friends (21.8%), social media (68.3%), and other sources (29.6%), which primarily consisted of their own research from the internet, books, journals, and from online endometriosis societies.

## Discussion

Prior to this study, there were no previous publications regarding patient awareness, knowledge, or understanding of preoperative imaging for DE. However, the findings of this study highlight patients’ lack of awareness or adequate education regarding the imaging modalities available for a preoperative diagnosis of endometriosis. Specifically, participants agreed that DE ultrasound, MRI, and CT can detect Stages I and II (namely, superficial endometriosis) in 55%, 45%, and 23.3% of cases, respectively (Figure 1 and Table 3), with the findings being similar when responses were stratified by the country of origin (Supplementary Table S2). This is in stark contrast to the current evidence in the literature, with ultrasound having low sensitivity for the detection of superficial endometriosis, ranging from 4.0% to 43.5% (20) to 65% (95% CI, 27%–100%) (21). The performance of MRI was similarly poor (22). As far as CT was concerned, no studies reviewed its performance, presumably due to its ineffectiveness in diagnosing small (<5 mm), soft-tissue lesions.

Participant responses and awareness regarding the use of DE ultrasound (36.6%), MRI (40.5%), and CT (23.2%) for the preoperative detection of bowel nodules also contrasted with those of the current literature. In a systematic review by Gerges et al. (10), the overall pooled sensitivity and specificity for DE ultrasound were 89% (95% CI, 83%–92%) and 97% (95% CI, 95%–98%), for MRI, they were 86% (95% CI, 79%–91%) and 96% (95% CI, 94%–97%), and for CT, they were 93% (95% CI, 84%–97%) and 95% (95% CI, 81%–99%). Similarly, while Stage IV endometriosis can encompass obliteration of the pouch of Douglas and DE nodules in multiple locations, including the rectosigmoid, torus uterinus, uterosacral ligaments, rectovaginal septum, bladder, and ureters, the participant responses revealed limited awareness of the effectiveness of ultrasound, MRI, and CT for preoperative diagnosis, reported at 54.9%, 45.8%, and 40.8%, respectively (Figure 1 and Table 3). These percentages are significantly lower than those reported on the performance of DE ultrasound and MRI, because these locations were based on different meta-analyses (10–12) and the recent international consensus statement on non-invasive imaging techniques for the diagnosis of pelvic DE (13–17).

Unsurprisingly, there was limited participant awareness of the role of preoperative imaging, particularly DE ultrasound and MRI, for the detection of ovarian endometriomas, with the responses being 63.4% and 61.3%, respectively (Figure 1 and Table 3). Once again, these figures contrast with those in the literature, with the sensitivity and specificity of ultrasound for the detection of endometriomas being 93%–95% and 96%–100% (20), respectively. However, the sensitivity and specificity of MRI were 95% (95% CI: 95%–100%) and 91% (95% CI: 86%–97%) (21, 22), respectively.

Although the majority of participants were from Australia (66.2%), followed by Canada (16.9%) and NZ (11.3%), there were more participants from Australia who were referred for a DE scan (48.9% vs. 25% vs. 12.5%, respectively), although the majority of those referred in all three countries proceeded to have the DE scan. It is difficult to ascertain the reasons for the higher referral rates in Australia, although they may be attributed to the mix of both private and public care and possibly to the fact that the procedure is referred to and performed in the private sector. However, it is impossible to determine this from this online survey, as this was not a question that was previously considered. There are certainly differences between countries with regard to reimbursement, accessibility, and education, which would undoubtedly impact the generalizability of the findings of this study.

The disparity between participant awareness in our study and the evidence in the literature is possibly due to misinformation from social media, given that 68.3% (Table 3) of participants identified social media as a source of knowledge. This is consistent with the findings of a study by Adler et al. (23), who analyzed evidence-based content on endometriosis posted on Instagram to find that only 36.8% of claims regarding diagnosis were evidence-based. They postulated that people did not consider other forms of diagnosis for endometriosis to be possible or accurate, given the common claim that “endo[metriosis] can only officially be diagnosed through invasive and expensive laparoscopic surgery or biopsy” (23). Furthermore, it was possible that patients were misinformed that they did not have endometriosis in the context of a “negative” scan, as would be the case with superficial endometriosis, only to be diagnosed at surgery. This further propagates that surgery is the only way to diagnose endometriosis. These findings serve to remind and encourage clinicians and researchers to publish evidence-based educational content on social media platforms, enriching public health communication, thereby strengthening the value of clinical translation.

Although this study is, to our knowledge, the first to assess patient awareness of preoperative imaging for the diagnosis of endometriosis, it is not without limitations. The main limitation pertains to the nature of an online survey, during which there is little control over factors such as the accuracy and validity of participants' understanding of the survey questions and their answers. Specifically, there is the issue of potential selection bias (24), as participants who join online endometriosis support groups are not representative of the broader patient population, as they are typically more engaged, symptomatic, and possibly have different levels of knowledge. This is further impacted by non-response bias (25), low-quality data (26, 27), and validation challenges (28). Diagnostic accuracy can be impacted because of the potential for self-reported diagnosis bias, with 38% of participants being unclear of their disease stage. Although the participants had to actively agree to proceed with the survey, there were no measures to prevent or detect fraudulent responses (e.g., CAPTCHAs and attention checks), which increases this risk (27). Also, for reasons that we are unable to explain, the response rates were significantly lower than we expected, possibly due to the survey potentially being perceived as irrelevant or not exciting enough for participants to warrant involvement. The results were heavily skewed, with the majority of the participants hailing from Australia and with minimal to no participants from Southeast Asia. This geographic bias significantly limits the generalizability of our findings, as the availability, cost, and clinician referral patterns for advanced imaging can vary dramatically by country. These limitations may be reduced in future studies with the incorporation of multilingual, multicenter prospective surveys while verifying clinical diagnosis through medical records to offset the cross-sectional self-report bias.

## Conclusion

This study highlights the lack of awareness and misinformation of patients with endometriosis regarding the availability and accuracy of preoperative imaging for the diagnosis of DE. This is a disappointing revelation for us as health professionals and experts in the field and stresses the importance of ensuring that accurate and understandable information is properly disseminated. The findings of this study also emphasize the need for the implementation of clinical education strategies to enhance practical value, which could include patient-friendly, standardized imaging brochures for gynecology clinics, formalized physician education protocols, and regulation of unsubstantiated medical content, while also using verified healthcare social media platforms for evidence-based dissemination. Furthermore, in the context of the recent international consensus statement on preoperative imaging and diagnosis (14), further studies are needed to assess the awareness of clinicians regarding this statement and how to bridge any gaps in this regard.

### Key message

The evidence for preoperative imaging for the diagnosis of deep endometriosis is well established; however, little is known about patient awareness regarding this evidence. This study highlighted that there continues to be a lack of patient awareness with regard to the availability and accuracy of preoperative imaging, specifically deep endometriosis ultrasound and magnetic resonance imaging, for the diagnosis of deep endometriosis. There is an increasing urgency for clinicians to address these knowledge gaps.

## Data Availability

The raw data supporting the conclusions of this article will be made available by the authors, without undue reservation.
