# Supplementary material for: Preoperative imaging for endometriosis awareness: an online survey study
Source: Front Reprod Health. 2026 Sep 3;8:1917458. doi: 10.3389/frph.2026.1917458 (PMC13582178; doi:10.3389/frph.2026.1917458)
Supplement: Supplementary file 1 [file Datasheet1.pdf]

Pre-Operative Imaging For Endometriosis Awareness Study: An Online Survey

Age ______________________

Relationship Status

◯ Single ◯ Engaged ◯ Married ◯ De Facto ◯ Separated ◯ Divorced ◯ Widowed
◯ Prefer not to say ◯ Other ______

Country of origin

◯ Australia ◯ Brazil ◯ Canada ◯ Spain ◯ UK ◯ USA ◯ Other ______

What is your highest level of education?

◯ High school ◯ Graduate Certificate or Diploma ◯ Bachelors Degree ◯ Masters Degree
◯ PhD ◯ Other______

Have you been previously diagnosed with endometriosis by a medical professional? ◯ Yes ◯ No

How long ago, in years, were you diagnosed with endometriosis? ______________________

What symptoms have you been experiencing? (Choose all that apply)

☐ Period pain ☐ Pelvic pain ☐ Pain with intercourse ☐ Bowel symptoms (e.g. difficulty or pain with bowel motions, bleeding) ☐ Bladder symptoms (e.g. difficult or pain emptying your bladder)
☐ Bloating ☐ Infertility ☐ Other______

Are you concerned about, or have you had any difficulties with getting pregnant?

◯ Yes ◯ No

Have you previously had IVF?

◯ Yes ◯ No

Have you been trying to fall pregnant for longer than six months?

◯ Yes ◯ No

On a scale of 0 to 10 how worried are you about endometriosis affecting your fertility?

Not worried at all — Extremely worried

(Place a mark on the scale above)

How were you diagnosed with endometriosis? (Choose all that apply)

☐ Based on symptoms ☐ Surgery (e.g. laparoscopy) ☐ Ultrasound ☐ MRI ☐ Other______

What treatments have you tried for endometriosis? (Choose all that apply)

☐ Herbal/Natural therapies ☐ Diet ☐ Non-steroidal anti-inflammatories ((e.g. ibuprofen, naprogesic, ponstan, etc) ☐ Oral contraceptive pills ☐ Progesterone only pills ☐ Ryeqo ☐ Zoladex ☐ Implanon ☐ Mirena intra-uterine device ☐ Surgery/Laparoscopy ☐ Other______
☐ I haven't tried anything

Have you had surgery for endometriosis?

◯ Yes ◯ No

If so, how many procedures have you had?

____

What stage of endometriosis were you diagnosed with?

◯ Stage 1 (Minimal) ◯ Stage 2 (Mild) ◯ Stage 3 (Moderate) ◯ Stage 4 (Severe) ◯ Unsure / I wasn't told

Did you have any of these investigations for your diagnosis of endometriosis or before your surgery? (Choose all that apply)

☐ Blood test (e.g. Ca125) ☐ Ultrasound ☐ MRI ☐ Deep endometriosis ultrasound ☐ CT
☐ Other______

Can endometriosis be diagnosed without surgery?

◯ Yes ◯ No

Can bowel endometriosis be diagnosed before surgery?

◯ Yes ◯ No

If so, how? (Choose all that apply)

☐ Ultrasound ☐ MRI ☐ CT scan ☐ Colonoscopy ☐ Other______

Have you ever been referred for a deep endometriosis ultrasound?

◯ Yes ◯ No

Did you go ahead getting the deep endometriosis ultrasound?

◯ Yes ◯ No

If not, why not? (Choose all that apply)

☐ Not available near me
☐ Too expensive
☐ Not convinced it was necessary
☐ Other______

If you had a deep endometriosis ultrasound or MRI before your surgery, how did you find out about it?

☐ Gynaecologist ☐ Fertility Specialist ☐ Family ☐ Friend ☐ Social Media ☐ Other______
☐ I did not have either before surgery

Deep Endometriosis Ultrasound can diagnose (Choose all that apply):

☐ Stage I (Minimal) endometriosis ☐ Stage II (Mild) endometriosis ☐ Stage III (Moderate) endometriosis
☐ Stage IV (Severe) endometriosis ☐ Bowel nodules ☐ Endometriomas (Endometriosis ovarian cysts)
☐ Adenomyosis (Endometriosis-like cells in the muscle of the uterus) ☐ Other______

MRI can diagnose (Choose all that apply):

☐ Stage I (Minimal) endometriosis ☐ Stage II (Mild) endometriosis ☐ Stage III (Moderate) endometriosis
☐ Stage IV (Severe) endometriosis ☐ Bowel nodules ☐ Endometriomas (Endometriosis ovarian cysts)
☐ Adenomyosis (Endometriosis-like cells in the muscle of the uterus) ☐ Other______

CT can diagnose (Choose all that apply):

☐ Stage I (Minimal) endometriosis ☐ Stage II (Mild) endometriosis ☐ Stage III (Moderate) endometriosis
☐ Stage IV (Severe) endometriosis ☐ Bowel nodules ☐ Endometriomas (Endometriosis ovarian cysts)
☐ Adenomyosis (Endometriosis-like cells in the muscle of the uterus) ☐ Other______

Where does your knowledge on endometriosis investigations and treatment come from?

☐ General Practioner (GP) ☐ Gynaecologist ☐ Fertility Specialist ☐ Family ☐ Friends
☐ Social Media (e.g. Instagram, TikTok, Facebook, etc) ☐ Other______
