# Supplementary material for: Preoperative imaging for endometriosis awareness: an online survey study
Source: Front Reprod Health. 2026 Sep 3;8:1917458. doi: 10.3389/frph.2026.1917458 (PMC13582178; doi:10.3389/frph.2026.1917458)
Supplement: Supplementary file 2 [file Table1.docx]

**Supplementary Table S1. Details of endometriosis history by country**

| **Variable** | **Australia N=94 (%)** | **Canada N=24 (%)** | **NZ N=16 (%)** | **Other N=8 (%)** |
| --- | --- | --- | --- | --- |
| **Relationship status** | | | | |
| Single | 33 (35.1) | 8 (33.3) | 5 (31.3) | 1 (12.5) |
| Engaged | 8 (8.5) | 2 (8.3) | 1 (6.3) | 2 (25) |
| Married | 26 (27.7) | 10 (41.7) | 2 (12.5) | 3 (37.5) |
| De Facto | 26 (27.7) | 0 (0) | 7 (43.8) | 2 (25) |
| Separated | 0 (0) | 2 (8.3) | 0 (0) | 0 (0) |
| Divorced | 0 (0) | 1 (4.2) | 0 (0) | 0 (0) |
| Other | 1 (1.1) | 1 (4.2) | 1 (6.3) | 0 (0) |
| **Highest level of education** | | | | |
| High school | 15 (16) | 7 (29.2) | 6 (37.5) | 1 (12.5) |
| Graduate Certificate or Diploma | 27 (28.7) | 6 (25) | 3 (18.8) | 0 (0) |
| Bachelors Degree | 33 (35.1) | 7 (29.2) | 5 (31.3) | 6 (75) |
| Masters degree | 17 (18.1) | 3 (12.5) | 2 (12.5) | 0 (0) |
| PhD | 0 (0) | 0 (0) | 0 (0) | 1 (12.5) |
| Other | 2 (2.1) | 1 (4.2) | 0 (0) | 0 (0) |
| Previously diagnosed with endo = Yes | 90 (95.7) | 21 (87.5) | 16 (100) | 7 (87.5) |
| **Symptoms (multiple allowed)** | | | | |
| Period pain | 87 (92.6) | 21 (87.5) | 15 (93.8) | 6 (75) |
| Pelvic pain | 89 (94.7) | 22 (91.7) | 16 (100) | 8 (100) |
| Pain with intercourse | 71 (75.5) | 14 (58.3) | 13 (81.3) | 5 (62.5) |
| Bowel symptoms | 79 (84) | 22 (91.7) | 14 (87.5) | 6 (75) |
| Bladder symptoms | 56 (59.6) | 13 (54.2) | 14 (87.5) | 4 (50) |
| Bloating | 87 (92.6) | 21 (87.5) | 15 (93.8) | 4 (50) |
| Infertility | 15 (16) | 10 (41.7) | 4 (25) | 2 (25) |
| Other | 22 (23.4) | 7 (29.2) | 4 (25) | 5 (62.5) |
| Concerned about or have had troubles with getting pregnant = Yes | 52 (55.3) | 11 (45.8) | 7 (43.8) | 3 (37.5) |
| Previous IVF = Yes | 4 (4.3) | 3 (12.5) | 1 (6.3) | 2 (25) |
| Trying to fall pregnant for longer than six months = Yes | 13 (13.8) | 5 (20.8) | 2 (12.5) | 2 (25) |
| **Diagnosis method (multiple allowed)** | | | | |
| Based on symptoms | 29 (30.9) | 11 (45.8) | 5 (31.3) | 2 (25) |
| Surgery | 72 (76.6) | 13 (54.2) | 14 (87.5) | 3 (37.5) |
| Ultrasound | 37 (39.4) | 8 (33.3) | 5 (31.3) | 4 (50) |
| MRI | 11 (11.7) | 3 (12.5) | 2 (12.5) | 0 (0) |
| Other | 8 (8.5) | 4 (16.7) | 0 (0) | 0 (0) |
| **Treatments tried (multiple allowed)** | | | | |
| Herbal=natural therapies | 40 (42.6) | 17 (70.8) | 11 (68.8) | 2 (25) |
| Diet | 60 (63.8) | 19 (79.2) | 14 (87.5) | 6 (75) |
| NSAIDs | 82 (87.2) | 18 (75) | 16 (100) | 5 (62.5) |
| Oral contraceptive pills | 56 (59.6) | 17 (70.8) | 13 (81.3) | 6 (75) |
| Progesterone only pills | 40 (42.6) | 12 (50) | 10 (62.5) | 3 (37.5) |
| Ryeqo | 7 (7.4) | 0 (0) | 1 (6.3) | 0 (0) |
| Zoladex | 5 (5.3) | 1 (4.2) | 1 (6.3) | 0 (0) |
| Implanon | 17 (18.1) | 2 (8.3) | 0 (0) | 1 (12.5) |
| Mirena intra-uterine device | 47 (50) | 9 (37.5) | 13 (81.3) | 1 (12.5) |
| Surgery | 70 (74.5) | 11 (45.8) | 14 (87.5) | 1 (12.5) |
| Other | 8 (8.5) | 5 (20.8) | 3 (18.8) | 0 (0) |
| I haven’t tried anything | 2 (2.1) | 0 (0) | 0 (0) | 2 (25) |
| Surgery for endometriosis = Yes | 73 (77.7) | 11 (45.8) | 14 (87.5) | 1 (12.5) |
| **Endometriosis stage** | | | | |
| Stage 1 | 5 (5.3) | 1 (4.2) | 0 (0) | 1 (12.5) |
| Stage 2 | 13 (13.8) | 4 (16.7) | 3 (18.8) | 1 (12.5) |
| Stage 3 | 15 (16) | 2 (8.3) | 6 (37.5) | 0 (0) |
| Stage 4 | 27 (28.7) | 7 (29.2) | 3 (18.8) | 0 (0) |
| Unsure = I wasn’t told | 34 (36.2) | 10 (41.7) | 4 (25) | 6 (75) |

*Participants could select more than one response where indicated.*  *CT, computed tomography; GP, general practitioner; MRI, magnetic resonance imaging; NSAIDs, non-steroidal anti-inflammatory drugs; NZ, New Zealand.*

**Supplementary Table S2. Patient understanding of pre-operative imaging for the diagnosis of deep endometriosis by country**

| **Variable** | **Australia N=94 (%)** | **Canada N=24 (%)** | **NZ N=16 (%)** | **Other N=8 (%)** |
| --- | --- | --- | --- | --- |
| **Pre-op investigations (multiple allowed)** | | | | |
| Blood test | 42 (44.7) | 12 (50) | 1 (6.3) | 1 (12.5) |
| Ultrasound | 89 (94.7) | 20 (83.3) | 16 (100) | 6 (75) |
| MRI | 28 (29.8) | 10 (41.7) | 2 (12.5) | 1 (12.5) |
| Deep endo ultrasound | 38 (40.4) | 3 (12.5) | 2 (12.5) | 0 (0) |
| CT | 19 (20.2) | 5 (20.8) | 2 (12.5) | 1 (12.5) |
| Other | 4 (4.3) | 1 (4.2) | 0 (0) | 2 (25) |
| Can endo be diagnosed without surgery = Yes | 48 (51.1) | 15 (62.5) | 8 (50) | 5 (62.5) |
| Can bowel endo be diagnosed before surgery = Yes | 32 (34) | 9 (37.5) | 2 (12.5) | 3 (37.5) |
| **Bowel endo diagnosis method (multiple allowed)** | **N=32** | **N=9** | **N=2** | **N=3** |
| Ultrasound | 21 (65.6) | 7 (77.8) | 2 (100) | 2 (66.7) |
| MRI | 23 (71.9) | 7 (77.8) | 2 (100) | 3 (100) |
| CT scan | 9 (28.1) | 4 (44.4) | 0 (0) | 2 (66.7) |
| Colonoscopy | 8 (25) | 3 (33.3) | 1 (50) | 3 (100) |
| Other | 1 (3.1) | 0 (0) | 0 (0) | 0 (0) |
| Referred for a deep endo ultrasound = Yes | 46 (48.9) | 6 (25) | 2 (12.5) | 2 (25) |
| Got deep endo ultrasound = Yes | 45 (97.8) N=46 | 5 (83.3) N=6 | 2 (100) N=2 | 1 (50) N=2 |
| Reasons why no deep endo ultrasound (multiple allowed) | N=1 | N=1 | N=0 | N=1 |
| Not available near me | 0 (0) | 0 (0) | NA | 0 (0) |
| Too expensive | 1 (100) | 0 (0) | NA | 1 (100) |
| Not convinced it was necessary | 0 (0) | 0 (0) | NA | 0 (0) |
| Other | 0 (0) | 1 (100) | NA | 0 (0) |
| **Found out about deep endometriosis ultrasound via (multiple allowed)** | | | | |
| Gynaecologist | 40 (42.6) | 5 (20.8) | 4 (25) | 1 (12.5) |
| Fertility specialist | 4 (4.3) | 1 (4.2) | 0 (0) | 0 (0) |
| Family | 1 (1.1) | 0 (0) | 0 (0) | 0 (0) |
| Friend | 3 (3.2) | 0 (0) | 0 (0) | 0 (0) |
| Social media | 6 (6.4) | 3 (12.5) | 1 (6.3) | 0 (0) |
| Other | 12 (12.8) | 1 (4.2) | 0 (0) | 1 (12.5) |
| I did not have either before surgery | 41 (43.6) | 14 (58.3) | 12 (75) | 6 (75) |
| **Deep endo ultrasound can diagnose (multiple allowed)** | | | | |
| Stage I | 24 (25.5) | 8 (33.3) | 3 (18.8) | 3 (37.5) |
| Stage II | 28 (29.8) | 7 (29.2) | 2 (12.5) | 3 (37.5) |
| Stage III | 37 (39.4) | 9 (37.5) | 6 (37.5) | 3 (37.5) |
| Stage IV | 54 (57.4) | 10 (41.7) | 11 (68.8) | 3 (37.5) |
| Bowel nodules | 37 (39.4) | 8 (33.3) | 5 (31.3) | 2 (25) |
| Endometriomas | 59 (62.8) | 14 (58.3) | 11 (68.8) | 6 (75) |
| Adenomyosis | 36 (38.3) | 10 (41.7) | 7 (43.8) | 3 (37.5) |
| Other | 22 (23.4) | 7 (29.2) | 2 (12.5) | 2 (25) |
| **MRI can diagnose (multiple allowed)** | | | | |
| Stage I | 18 (19.1) | 5 (20.8) | 2 (12.5) | 4 (50) |
| Stage II | 19 (20.2) | 7 (29.2) | 4 (25) | 5 (62.5) |
| Stage III | 32 (34) | 10 (41.7) | 4 (25) | 3 (37.5) |
| Stage IV | 40 (42.6) | 13 (54.2) | 8 (50) | 4 (50) |
| Bowel nodules | 35 (37.2) | 12 (50) | 7 (43.8) | 4 (50) |
| Endometriomas | 55 (58.5) | 14 (58.3) | 12 (75) | 6 (75) |
| Adenomyosis | 30 (31.9) | 11 (45.8) | 8 (50) | 4 (50) |
| Other | 25 (26.6) | 6 (25) | 2 (12.5) | 2 (25) |
| **CT can diagnose (multiple allowed)** | | | | |
| Stage I | 11 (11.7) | 2 (8.3) | 2 (12.5) | 3 (37.5) |
| Stage II | 10 (10.6) | 1 (4.2) | 1 (6.3) | 3 (37.5) |
| Stage III | 11 (11.7) | 2 (8.3) | 2 (12.5) | 2 (25) |
| Stage IV | 22 (23.4) | 3 (12.5) | 5 (31.3) | 3 (37.5) |
| Bowel nodules | 21 (22.3) | 4 (16.7) | 4 (25) | 4 (50) |
| Endometriomas | 47 (50) | 9 (37.5) | 7 (43.8) | 5 (62.5) |
| Adenomyosis | 20 (21.3) | 4 (16.7) | 3 (18.8) | 3 (37.5) |
| Other | 34 (36.2) | 11 (45.8) | 6 (37.5) | 2 (25) |
| **Where is knowledge on endometriosis investigations from (multiple allowed)** | | | | |
| GP | 64 (68.1) | 6 (25) | 8 (50) | 3 (37.5) |
| Gynaecologist | 74 (78.7) | 11 (45.8) | 13 (81.3) | 4 (50) |
| Fertility specialist | 11 (11.7) | 3 (12.5) | 1 (6.3) | 1 (12.5) |
| Family | 11 (11.7) | 5 (20.8) | 1 (6.3) | 0 (0) |
| Friends | 21 (22.3) | 6 (25) | 2 (12.5) | 2 (25) |
| Social media | 58 (61.7) | 20 (83.3) | 14 (87.5) | 5 (62.5) |
| Other | 30 (31.9) | 5 (20.8) | 3 (18.8) | 4 (50) |

*Participants could select more than one response where indicated. CT, computed tomography; GP, general practitioner; MRI, magnetic resonance imaging; NSAIDs, non-steroidal anti-inflammatory drugs; NZ, New Zealand*

**Appendix**

**Appendix 1. Survey questions**
